# Gedunin and Azadiradione: Human Pancreatic Alpha-Amylase Inhibiting Limonoids from Neem (*Azadirachta indica*) as Anti-Diabetic Agents

**DOI:** 10.1371/journal.pone.0140113

**Published:** 2015-10-15

**Authors:** Sudha Ponnusamy, Saikat Haldar, Fayaj Mulani, Smita Zinjarde, Hirekodathakallu Thulasiram, Ameeta RaviKumar

**Affiliations:** 1 Institute of Bioinformatics and Biotechnology, Savitribai Phule Pune University, Pune 411 007, Maharashtra, India; 2 Chemistry-Biology Unit, Division of Organic Chemistry, CSIR-National Chemical Laboratory, Pune 411 008, Maharashtra, India; Weizmann Institute of Science, ISRAEL

## Abstract

Human pancreatic *α*-amylase (HPA) inhibitors offer an effective strategy to lower postprandial hyperglycemia via control of starch breakdown. Limonoids from *Azadirachta indica* known for their therapeutic potential were screened for pancreatic α-amylase inhibition, a known anti-diabetic target. Studies were carried out to reveal their mode of action so as to justify their hypoglycemic potential. Of the nine limonoids isolated/semi-synthesized from *A*.*indica* and screened for *α*-amylase inhibition, azadiradione and exhibited potential inhibition with an IC_50_ value of 74.17 and 68.38 μM, respectively against HPA under *in vitro* conditions. Further screening on AR42J *α*-amylase secretory cell line for cytotoxicity and bioactivity revealed that azadiradione and gedunin exhibited cytotoxicity with IC_50_ of 11.1 and 13.4μM. Maximal secreted *α*-amylase inhibition of 41.8% and 53.4% was seen at 3.5 and 3.3μM, respectively. Michaelis-Menten kinetics suggested a mixed mode of inhibition with maltopentaose (*K*_*i*_ 42.2, 18.6 μM) and starch (*K*_*i*_ *′* 75.8, 37.4 μM) as substrate with a stiochiometry of 1:1 for both azadiradione and gedunin, respectively. The molecular docking simulation indicated plausible π-alkyl and alkyl-alkyl interactions between the aromatic amino acids and inhibitors. Fluorescence and CD confirmed the involvement of tryptophan and tyrosine in ligand binding to HPA. Thermodynamic parameters suggested that binding is enthalpically and entropically driven with ΔG° of -21.25 kJ mol^-1^ and -21.16 kJ mol^-1^ for azadiradione and gedunin, respectively. Thus, the limonoids azadiradione and gedunin could bind and inactivate HPA (anti-diabetic target) and may prove to be lead drug candidates to reduce/control post-prandial hyperglycemia.

## Introduction

Diabetes mellitus (DM) is a metabolic disorder resulting from a defect in insulin secretion, insulin action, or both leading to chronic hyperglycemia. It is often accompanied with disturbances of carbohydrate, fat and protein metabolism and severe diabetic complications such as retinopathy, neuropathy, nephropathy, cardiovascular complications and ulceration [1–4]. WHO projects diabetes to be the 7^th^ leading cause of death afflicting up to 366 million globally with 79.4million individuals being affected by 2030 [5–7].An effective therapeutic approach for management of diabetes and obesity is to decrease hyperglycemia by retarding and reducing the digestion of ingested carbohydrates. Inhibition of carbohydrate degrading enzymes significantly reduces post prandial increase in blood glucose after a meal by delaying starch hydrolysis [8]. This suppression of post prandial hyperglycemia delays the progression of vascular complications associated with DM [9]. One such enzyme, human pancreatic α-amylase (HPA, α-1,4-glucan-4-glucanohydrolase, E.C. 3.2.1.1) plays a pivotal role in DM. It catalyses the initial step in hydrolysis of starch to maltose which is eventually degraded to glucose by α-glucosidases. Hence, retardation of starch digestion by HPA inhibition plays a key role in the control of post prandial hyperglycemia in type II DM [10,11]. By inhibiting HPA in the small intestines, the rate of hydrolysis of starch is decreased delaying the digestion process. This spreading of digestion process reduces the amount of glucose generated and released in the blood and is one of the effective strategies in lowering post prandial hyperglycemia.

A useful model system to study the inhibition of secreted HPA is the rat pancreatic acinarAR42J cell line, derived from azaserine-induced malignant nodules from rat pancreas. The cell line is an amphicrine model with exocrine and endocrine functions and is characterized by the presence of digestive enzyme-containing dense core vesicles [12]. Inducing the cell line with glucocorticoid dexamethasone converts pluripotent pancreatic AR42J cells into exocrine cells expressing these digestive enzymes by increasing the intracellular, secreted amylase contents and making the cell line an ideal system to work with pancreatic α-amylase inhibitors [13]. Loading these induced acinar cells with varying starch loads would mimic or simulate the physiological conditions. Only few reports on screening of compounds for α-amylase inhibition with cell line studies for bioactivity exist.

The currently available treatments have side effects such as hypoglycemia, weight gain and other complications which necessitate the need for development of new antidiabetic targets and therapies for glycemic control [14–16]. The inability of current therapies to control hyperglycemia without any side effects along with its high cost and poor availability impels the search towards traditional herbal remedies which may provide valuable leads and therapeutic strategies. Also HPA inhibitors have been reported to be devoid of side effects [17]. The use of natural plant products as a complementary approach for management of DM is growing with >1200 plants being reported to have anti-diabetic effects. The key obstacles which have restricted the utilization of alternative medicines are their lack of proper documentation, stringent quality control; identification of key bioactive components and their mechanism of action [18, 19]. Moreover, only a few comprehensive studies on scientific validation of traditional antidiabetic medicinal plants are known and thus offer an attractive source of HPA inhibitors.

**Fig 1:**
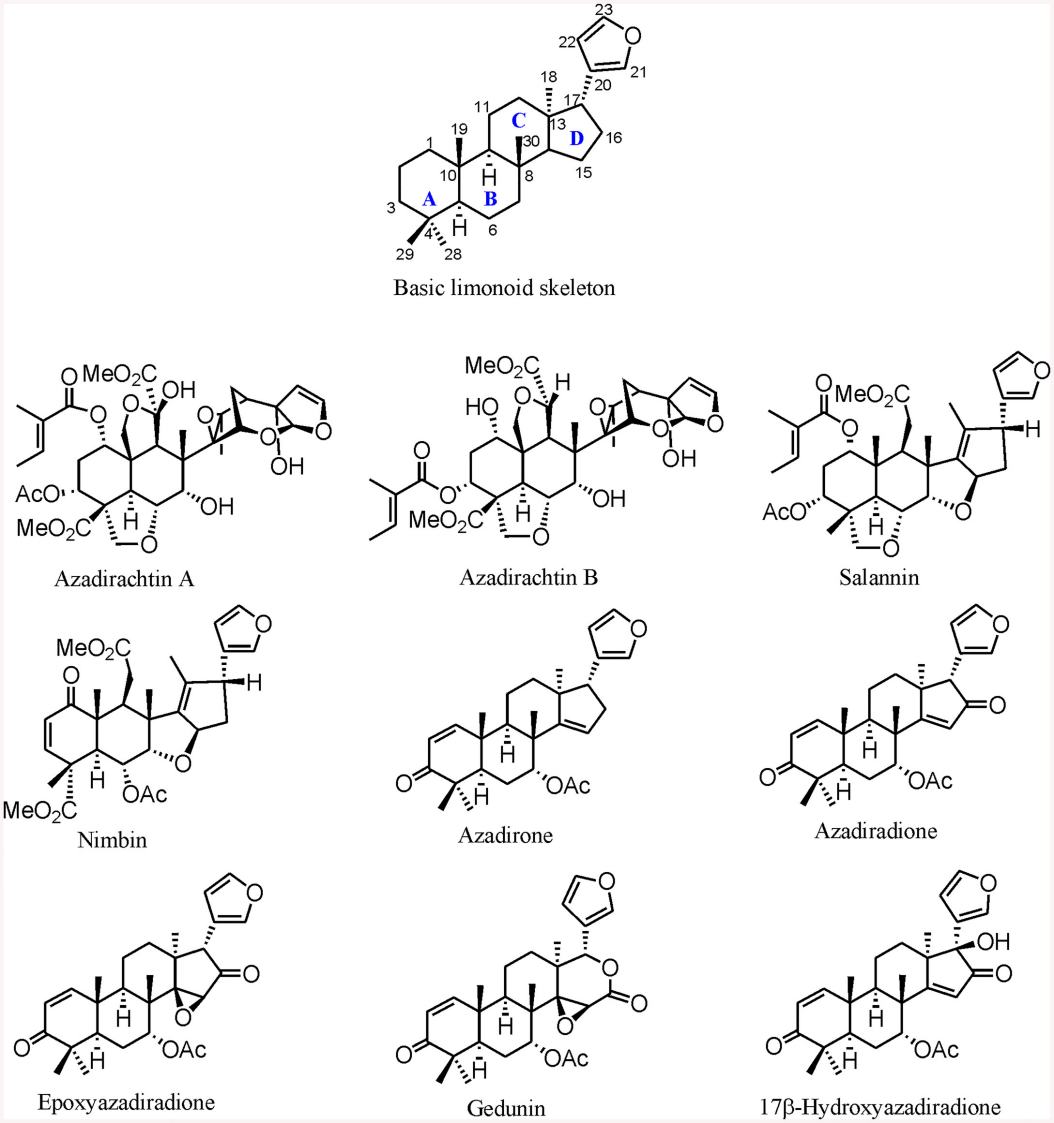
Basic limonoid structure and limonoids from *A*.*indica*.

The ‘*wonder tree*’ Neem (*Azadirachta indicia* A. Juss.; Meliaceae), native to Indian subcontinent but cultivated throughout the tropics is well-known for its diverse medicinal uses for more than 2000 years. Earlier studies have shown that the aqueous leaf extract of Neem resulted in hypoglycemia in normal rats and lowered blood sugar level in streptozotocin induced diabetic rats [20,21]. It is one of the richest known sources of secondary metabolites in nature, especially tetranortriterpenoids (limonoids). Over 150 skeletally diverse and oxygenated triterpenoids have been isolated and characterized from various parts of the Neem plant in last five decades and they have been investigated to possess a wide-spectrum of pharmacological activities and insecticidal potency [22,23]. Limonoids possess 4,4,8-trimethyl-17-furanylsteroidal skeleton which is further substituted with other functional groups (Fig 1). Neem limonoids can be classified skeletally into two groups; basic limonoids (4,4,8-trimethyl-17-furanylsteroidal skeleton such as azadirone, azadiradione, gedunin) and C-seco limonoids (with modified and rearranged C-ring such as azadirachtin, salannin, nimbin) [22,24].Very few studies on the tertranortriterpenoids effect on α-amylase are available. Recently, the tetranortriterpenoid meliacinolin and azadirachtolide isolated from *A*. *indica* leaves, and swietenine from *Swietenia macrophylla* have been reported to exhibit α-amylase inhibitory activity in streptozotocin induced diabetes in mice [25–27]. On this basis, the current study involves nine neem limonoids four of C-seco type [azadirachtin A, azadirachtin B, salannin and nimbin] and five containing basic limonoid skeleton [azadirone, azadiradione, epoxyazadiradione, gedunin and 17β-hydroxyazadiradione] isolated and characterized in our previous study [24, 28,29]. The goals of this study were (1) screening and identification of Neem limonoids as potent inhibitors of HPA on the basis of their inhibition potency and *in vitro* cytotoxicity on AR42J cell line (2) to reveal their mode of action and underlying molecular interactions with respect to molecular docking, inhibition kinetics and ligand binding.

## Materials and Methods

### Chemicals and reagents

Human pancreatic α-amylase (HPA), maltopentaose, were purchased from Sigma Aldrich, USA. 3,5-dinitrosalicylicacid (DNSA), Ham’s F-12K with L-glutamine culture media, fetal bovine serum, antibiotics streptomycin, penicillin, Trypsin (3-(4,5-Dimethylthiazol-2-yl)-2,5-diphenyltetrazoliumbromide (MTT) were obtained from HiMedia Laboratories, Mumbai, India. Porcine pancreatic α-amylase (PPA) was procured from SRL, Mumbai, India. AR42J rat pancreatic cell line was from ATCC-CRL142. All other chemicals and solvents from local manufacturer were of AR or HPLC grade.

### Neem limonoids

The chemical structure of neem limonoids isolated and characterized from Neem fruits (fruit coat/seed) are presented in Fig 1. We have previously reported the isolation and characterization of these compounds and no specific permissions were required for these isolations. Azadirachtin A, azadirachtin B, salannin, nimbin were isolated from Neem seed kernel whereas azadiradione, epoxyazadiradione, azadirone were purified from fruit coat. The purity of the compounds as determined by HPLC was 98.8, 99.1, 99.1, 98.2,98.9, 98.1 and 99.6%forazadirachtin A (retention time (*Rt*) 28.3 min), azadirachtin B (*Rt* 29.7 min), nimbin (*Rt* 47.9 min), salannin (*Rt* 52.2 min), azadiradione (*Rt* 46.0 min), azadirone (*Rt* 16.2 min) and epoxyazadiradione (*Rt* 56.2 min), respectively as mentioned in our earlier studies [24,28,29]. Structure of the isolated/semi-synthesized limonoids was confirmed on the basis of LC-electrospray ionization-MS and NMR (^1^H, ^13^C, DEPT-135) studies.

### Primary screening

The inhibition assay was performed using the chromogenic DNSA method [30,31]. PPA was used for preliminary screening of α-amylase inhibitors. According to Pasero et al (1986), the cDNA nucleotidic sequence alignment of mammalian amylases shows a high degree of similarity between porcine, human and mouse α-amylases [32]. Moreover a high degree gene sequence similarity (~ 90%) exists between human salivary α-amylase (HSA) and PPA as reported by (Buisson et al 1987) [33]. Brayer et al (1995) have reported 97% primary sequence similarity between human salivary and human pancreatic α-amylases and a 70% structural similarity between PPA and HPA [11]. Thus, a high degree of similarity between PPA and HPA allows us to use it as the enzyme for screening inhibitors.

In brief, the total assay mixture consisting of 0.02 M sodium phosphate buffer, pH6.9, (containing 6 mM NaCl), 0.2–0.25 U of PPA and inhibitors (13.8–214.3 μM) in DMSO was incubated at 37°C for 10 min. After pre-incubation, the substrate starch (1%, w/v) solution was added to the reaction mixture and further incubated for 15 min. The control PPA (0.21 U) without any inhibitor represented 100% enzyme activity. One unit of enzyme activity is defined as the amount of enzyme required to release one micromole of maltose from starch/maltopentaose per min under the assay conditions. The other quantifiers calculated were:

% Relative enzyme activity = (enzyme activity of test/enzyme activity of control)*100.

% inhibition = (100 − % relative enzyme activity).

The IC_50_ values were determined from plots of percent inhibition versus inhibitor concentration and calculated by logarithmic regression analysis. To eliminate the absorbance produced by the compound, appropriate controls were included. The known PPA/HPA inhibitor acarbose was used as a positive control at a concentration range of 10–50 μM. The compounds exhibiting PPA inhibition were subjected to docking simulations and for studies on AR42J cell line.

### Cytotoxicity and bioactivity in AR42J cell line

AR42J rat pancreatic acinar cells were used to screen the compounds exhibiting PPA inhibition for cytotoxicity and bioactivity. The cell lines were cultured in Ham’s F-12K with L-glutamine culture media supplemented with nonessential amino acids, sodium pyruvate, 7.5% sodium bicarbonate, 20% fetal bovine serum and antibiotics (100 μgml^-1^ streptomycin, 100 unitsml^-1^ penicillin) at 37°C under a humidified condition of 95% air and 5% CO_2_. Cells were seeded at 2×10^4^ cells well^-1^ onto 96 wells culture dish. After overnight attachment of the cells, the medium was replaced with the medium containing 10 nM dexamethasone, and the compounds at a concentration of 1.7–36.3 μM in 1% DMSO for 24 h [13]. Appropriate medium and DMSO controls were simultaneously set up. The amount of α-amylase released by cells was determined in an aliquot of medium by DNSA method. At the end of 24 h incubation period the culture media was removed from the samples and fresh culture media (100 μl) containing 20 μl of MTT tetrazolium bromide (5 mg ml^-1^) added and incubated for 3 h at 37°C. Metabolically active cells reduce MTT to insoluble purple formazan dye crystals, which were dissolved by addition of 300 μl DMSO solution at the end of the incubation period and quantitated at 550 nm [34]. In order to ascertain the efficiency of inhibitors in presence of physiological substrate starch, the secreted pancreatic α-amylase inhibition was determined by performing starch load test. Starch (0.25–1%) was added to the inhibitor treated cells and to the appropriate controls. Medium aliquot at 1hr, 2hr and 3 hr were removed from the wells for estimation of secreted enzyme activity by DNSA method.

### Docking simulations

Docking simulations of inhibitors with HPA (PDB ID: 1HNY), were performed using AutoDockVina [11, 35]. Standardized docking parameters, as obtained from the previous studies were used [36]. Blind docking and refine docking was done with the grid box spacing of 0.5 Å. A grid box encompassing the HPA molecule, of (106x126x126 points), and grid box center set at x = 8.499, y = 61.167, and z = 13.444 was used for blind docking for all the inhibitors, whereas refine docking simulation were done with grid parameters that scored high in blind docking. Grid box of 50x66x44 points and center set at x = 16.250, y = 42.528, and z = 15.583 for epoxyazadiradione, gedunin, whereas for azadiradione as a grid box of 74x48x40 points and center set at x = 5.582, y = 42.813, and z = 41.372. The interactions of inhibitor-HPA were analyzed and visualized in Discovery Studio 4.0 client [37].

### Human pancreatic α-amylase inhibition, stiochiometry and kinetics

The lead inhibitors azadiradione and gedunin, were subjected to HPA inhibition at concentrations exhibiting minimum cytotoxicity and maximal bioactivity. Inhibition assay was performed similar to that of PPA with 0.2–0.25 U of HPA and individual inhibitors azadiradione (22.2–133.3 μM), gedunin (20.7–124.3 μM). The control HPA (0.25 U) without inhibitors represented 100% enzyme activity. In order to demonstrate the stoichiometry of HPA inhibition, the enzyme (14.85μM) in buffer was added to a solution of varying concentrations of azadiradione (22.2–133.3 μM) and gedunin (20.7–124.3 μM), incubated for 10 min and assayed as mentioned above and percent inhibition determined.

The mode of inhibition of HPA by the lead inhibitors azadiradione and gedunin were determined using Michaelis-Menton and Lineweaver-Burk equations. Starch (1–5mg ml^-1^) and maltopentaose (0.05–0.4mM) were incubated with inhibitors-HPA for 10 and 2.5 min, respectively and the residual enzyme activity determined by DNSA method for starch and Nelson-Somogyi’s method for maltopentaose [38].The inhibitor constants(*K*_*i*_) were determined on the basis of mode of inhibition.

### Ligand binding studies

#### Fluorescence measurement

Ligand binding studies using fluorescence measurements of HPA were carried as described in our earlier report [34]. Briefly, HPA was titrated with azadiradione (3.9–118.1 μM) and gedunin (2.1–51.8 μM) followed by monitoring the change in fluorescence at 350 nm. Non-linear and linear regression analyses were used to analyze the data.

The thermodynamic parameters, association constants (*K*_*a*_), dissociation constant (*K*_*d*_) and Gibbs free energy (*ΔG°*) of binding for azadiradione and gedunin were determined at various temperatures 25°C to 60°C by fluorescence quenching studies.

#### Circular Dichroism (CD) spectroscopy

CD of HPA, in presence and absence of the ligand along with the appropriate controls of buffer blank and ligand blank were recorded in the near-uv (250–320 nm) and far–uv (195 to 250 nm) regions. The quartz cuvette (0.1-cm) contained 50–100 μg of HPA in 0.02 M sodium phosphate buffer (pH 6.9). For ligand binding analysis, ligands at the concentration exhibiting maximum bioactivity in the cell lines was pre-incubated along with HPA for 10 min and CD scans recorded.

#### Docking of ligands to PPA-substrate complex

Docking simulations of the ligand to the enzyme-substrate (maltopentaose) complex were performed to reveal the binding of limonoids in presence of substrate using Autodock Vina. The structural X-ray map of a PPA crystal soaked (and flash-frozen) with maltopentaose substrate (PDB: 1UA3) was taken as the enzyme-substrate complex [39] to dock azadiradione and gedunin. The structure of the co-crystal refined at 2.01Å to an *R* factor of 17% showed an electron density pattern corresponding to the binding of oligosaccharides at the active site and at three other surface binding sites. Blind docking, encompassing the PPA-oligosaccharide complex with the grid box, parameters (92x126x120 points), and grid box center set at x = 33.255, y = 30.824, and z = 14.790 with the grid box spacing of 0.5 Å for both azadiradione and gedunin were performed individually. The interactions of inhibitor-PPA-oligosaccharide were analyzed and visualized in Discovery Studio 4.0 client [37].

### Statistical analysis

Each experiment was repeated as 3 independent sets with each set in triplicates. Results are expressed as means S.E.M. with number of observations (n). The best-fit values were achieved by applying either linear fit or non-linear least square regression using the software, Microcal Origin 6.0 (Micarocal Software Inc., Northampton, USA). Data was analyzed by student’s t-test using SPSS statistical package SPSS 17.0(SPSS Inc., Chicago, IL, USA).Differences were considered statistically significant for p < 0.05.

## Results

### Primary screening

Porcine pancreatic α-amylase (PPA) an enzyme having a high degree of sequence, structural and functional similarity with HPA as mentioned earlier was used for preliminary screening of compounds possessing α-amylase inhibitory property [11,32,33]. A total of nine limonoids, namely, azadirachtin A, azadirachtin B, salannin, nimbin, azadirone, azadiradione, epoxyazadiradione, gedunin and 17β-hydroxyazadiradione from *A*. *indica* were screened for PPA inhibition. Limonoids azadiradione, epoxyazadiradione and gedunin exhibited PPA inhibition with IC_50_ values of 138.4, 100.2, and 72.2 μM, respectively. No inhibition was exhibited by other six limonoids *viz*., azadirachtin A, azadirachtin B, salannin, nimbin, azadirone and 17β-hydroxyazadiradione.

### Cytotoxicity and bioactivity in AR42J cell line

**Fig 2:**
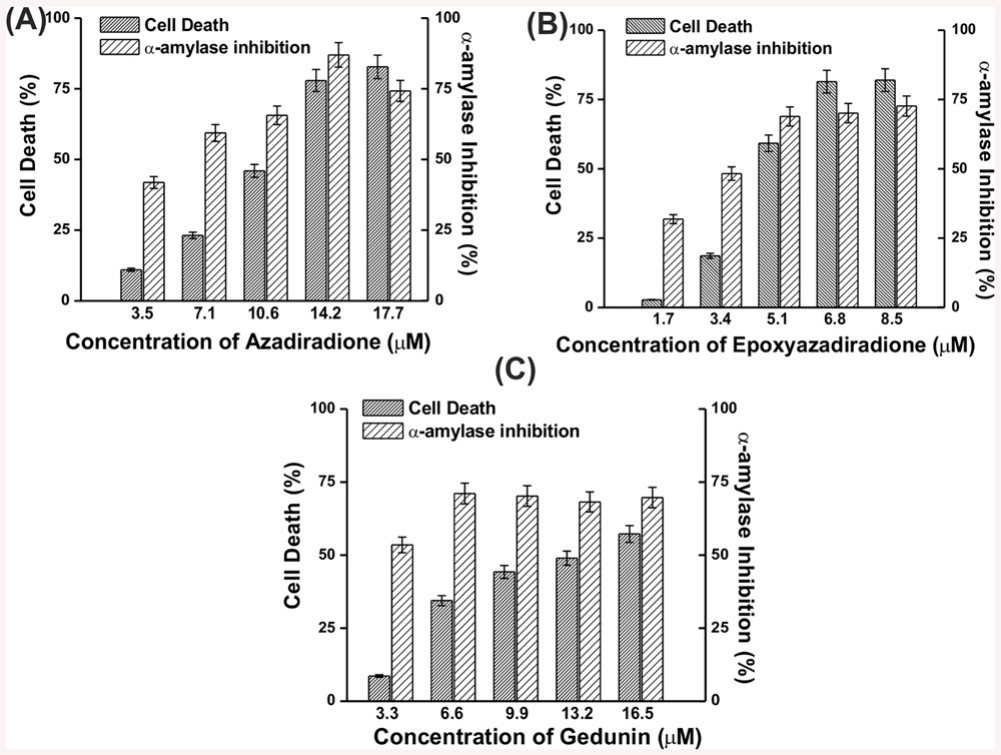
Cytotoxicity and pancreatic inhibition in AR42J cell line. Cytotoxicity and inhibition of secretory pancreatic α-amylase activity at varying concentrations of azadiradione (A), epoxyazadiradione (B) and gedunin (C) Error bars represent ±SE of the mean of triplicates and p values <0.05 were considered significant.

**Fig 3:**
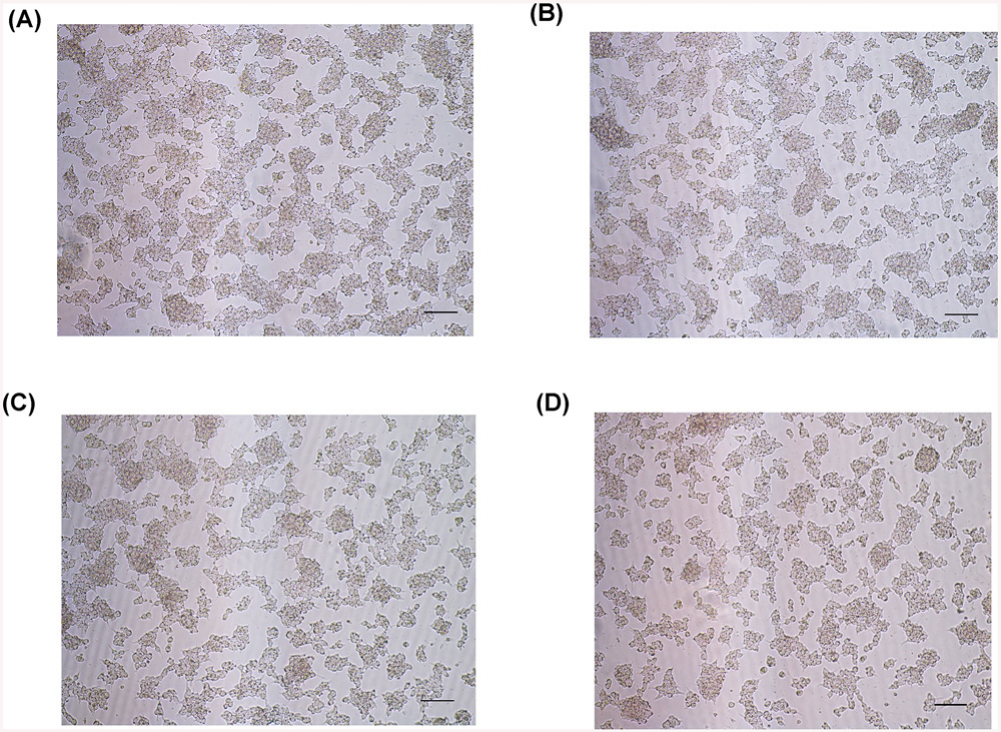
Effect of limonoids on AR42J cell morphology. Inverted phase contrast image under magnification of 100X of AR42J cell line treated under following conditions A) Control, B) Dexamethasone induced (10 nM), C) Azadiradione at 3.5 μM D) Gedunin at 3.3 μM.

Cytotoxicity and bioactivity of azadiradione (3.5–17.7 μM), epoxyazadiradione (1.7–8.5 μM) and gedunin (3.3–16.5 μM) on the amylase secretory cell line AR42J was carried out. These compounds were found to be cytotoxic at IC_50_ values of 11.1, 13.4 and 4.1 μM for azadiradione, gedunin and epoxyazadiradione, respectively while exhibiting amylase inhibition of 70.1, 69.2 and 53.2%. At concentrations of 3.5, 1.7 and 3.3 μM, lower cell death of 8.6, 2.7 and 8.6% with reasonably high pancreatic amylase inhibition of 41.8, 31.8 and 53.4%was seen for azadiradione, epoxyazadiradione and gedunin, respectively (Fig 2). The bioactivity in presence of varying starch loads (0.25–1%) which mimicked the physiological post-prandial conditions was also determined. Azadiradione and gedunin exhibited 22.9% and 31.7% secreted amylase inhibition at 0.5% (w/v) starch up to two hours. Moreover, no morphological change could be observed in azadiradione and gedunin treated cells under these conditions as compared to controls (Fig 3).

### Docking simulation

**Fig 4:**
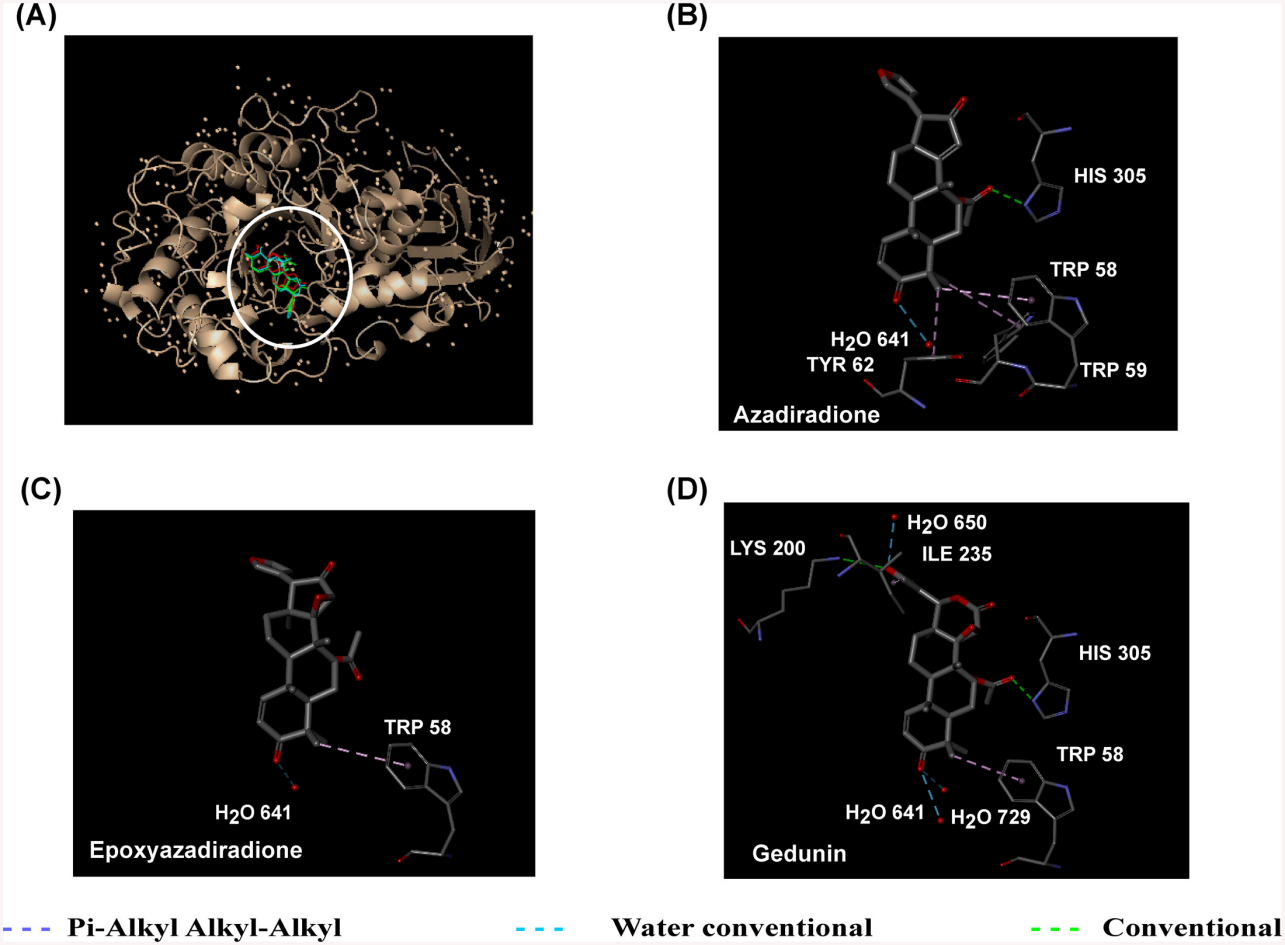
Docking of limonoids to HPA. Limonoids docked on HPA (A). Putative mode of interaction between azadiradione, epoxyazadiradione and gedunin from *A*. *indica* and with HPA (B, C, D).

*In silico* screening of Neem limonoids azadiradione, epoxyazadiradione and gedunin docked onto HPA was carried out with the validated docking parameters as reported in our earlier study [36]. Initial blind docking of all three compounds generated 90 poses each. Of these blind poses (35, 30, and 22%) possessing minimal energy for azadiradione, epoxyazadiradione, gedunin, respectively docked near the active pocket (Fig 4A). The subsequent refined docking of the compounds generated the best poses with minimal energy of interaction (-25.5, -33.4, and -25.9 kJ mol^-1^) for azadiradione, epoxyazadiradione and gedunin respectively. The interactions with azadiradione include pi-alkyl interaction between, Trp 58, Tyr 62 with C29 of A ring, and Trp 59 with C28 with bond lengths of 4.83, 4.94, and 4.84 Å, respectively. Conventional hydrogen bonding with a bond length of 3.12 Å was observed between oxygen of acetyl group at C7 position and His 305. The oxygen at C3 keto was observed to have a conventional hydrogen bonding with H_2_O 641 with a bond length of 3.2 Å. Epoxyazadiradione forms pi-alkyl interaction between, Trp 58 with C29 of A ring with a bond distance of 4.98 Å and a conventional hydrogen bond between C3 keto and H_2_O 641 with a bond distance of 3.28 Å. The principle interaction of gedunin with HPA involves pi-alkyl interaction between, Trp 58 and C29 methyl of A ring with a bond length of 5.30 Å. The five membered furan ring is also involved in the pi-alkyl interaction with Ile 235 and bond length 5.04 Å. Conventional hydrogen bond with a bond length of 3.19, 3.36, 3.08, 3.19, 3.22 Å, is observed between (C3 keto oxygen and H_2_O 641), (C3 keto oxygen and H_2_O 729), (acetoxy oxygen at C7 and His 305), (oxygen of the furan ring and Lys 200) and (oxygen of furan ring with H_2_O 650) respectively.

### HPA inhibition, stiochiometry and kinetics

**Fig 5:**
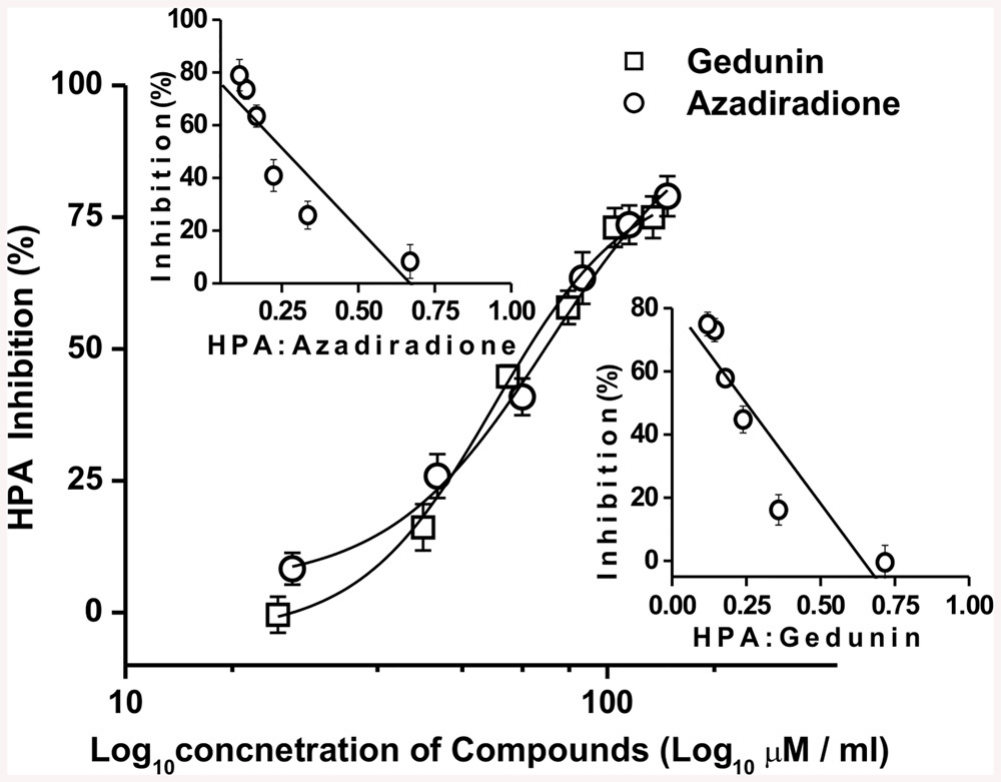
Effect of limonoids on HPA activity. Sigmoidal plot of HPA relative enzyme inhibition (%) versus varying concentration gedunin and azadiradione. Inset: stoichiometery of HPA inhibition by gedunin and azadiradione. Error bars represent ± SE of the mean of triplicates and p values < 0.05 were considered significant.

**Fig 6:**
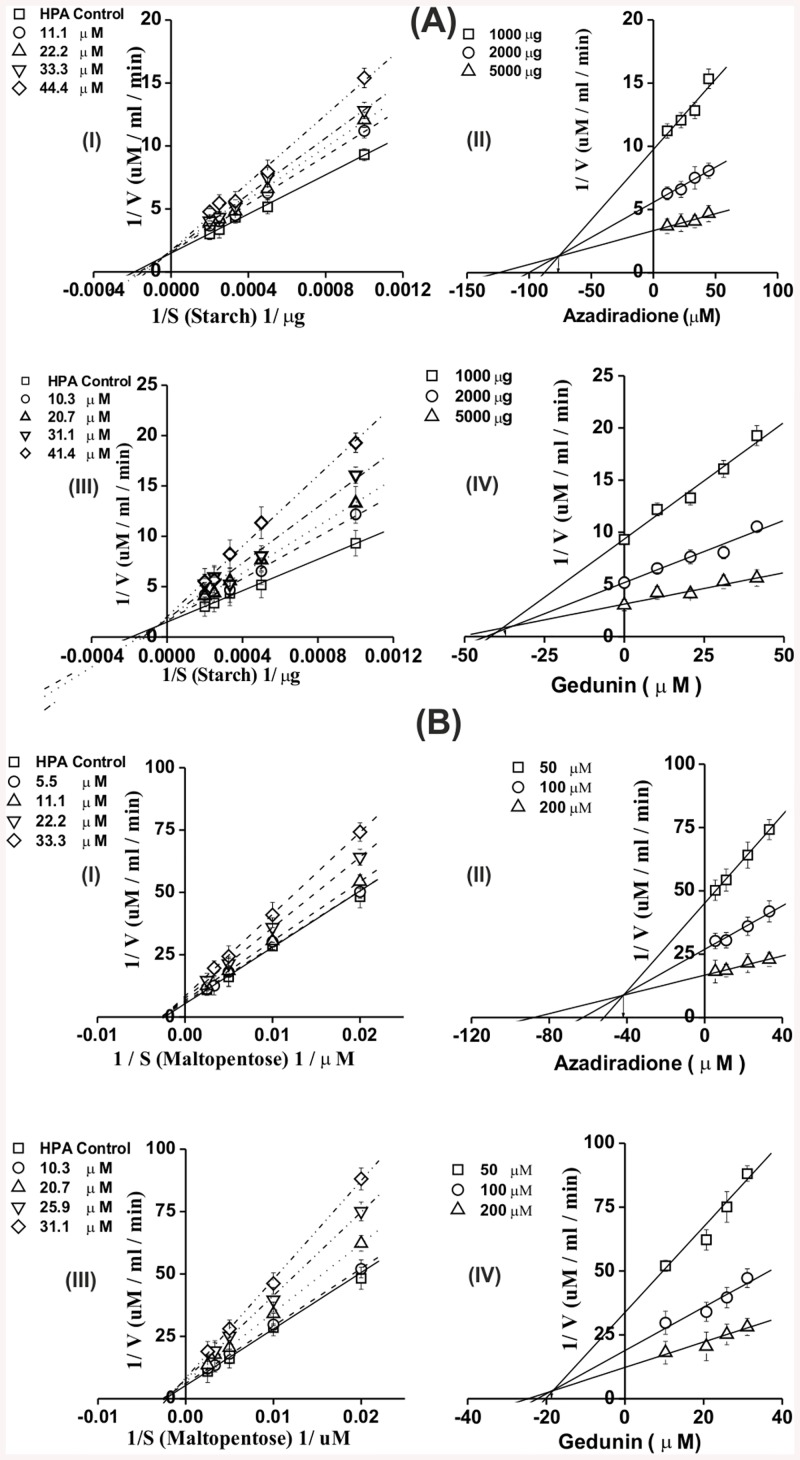
HPA inhibition kinetics with limonoids. LineWeaver-Burk plot and Secondary plot for HPA inhibition at varying azadiradione and gedunin concentrations with starch (A) and maltopentaose (B) as substrate. Error bars represent ±SE of the mean of triplicates and p values <0.05 were considered significant.

Based on their lower cytotoxicity and higher bioactivity values, the lead molecules azadiradione and gedunin were carried forward for further studies. A concentration dependent inhibition by azadiradione (22.2–133.1 μM) and gedunin (20.7–124.3 μM) was observed against HPA activity. As shown in Fig 5, azadiradione and gedunin inhibited HPA with sigmoidal fits and exhibited an IC_50_ value of (74.17 and 68.38 μM) respectively. Acarbose used as a positive control, showed an IC_50_ value of 15 μM against HPA. The stiochiometry of inactivation of HPA with azadiradione and gedunin is shown in Fig 5 inset. On plotting percentage inhibition versus molar ratio of HPA and inhibitor, a stoichiometry of 0.66 and 0.68 were obtained for azadiradione and gedunin respectively indicating 1:1 stoichiometric interaction between enzyme and ligand. The mode of HPA inhibition was kinetically determined at differing inhibitor concentration using starch and maltopentaose as substrates. The double reciprocal Lineweaver–Burk plots revealed that the mode of inhibition to be mixed for both azadiradione and gedunin with the substrates starch and maltopentaose (Fig 6). The inhibitor constant *K*_*i*_ determined using secondary plots suggested an apparent *K*_*i*_ *′* of 75.8 and 37.4 μM for starch as substrate and *K*_*i*_ of 42.2 and 18.6 μM with maltopentaose for azadiradione and gedunin, respectively.

### Ligand binding

#### Fluorescence studies

**Fig 7:**
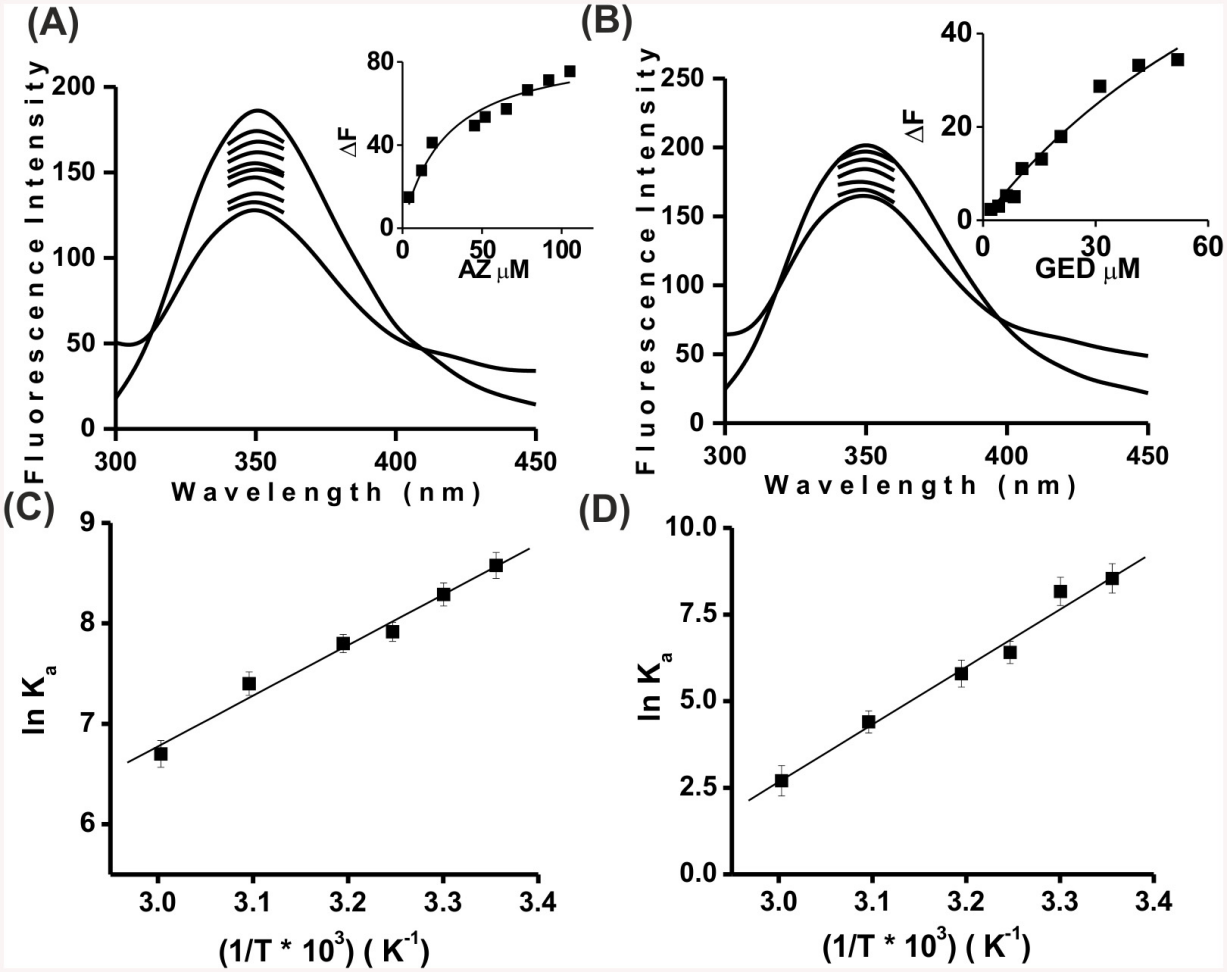
Ligand binding studies by fluorescence. Quenching of intrinsic fluorescence. (A) Azadiradione bound HPA. (B) Gedunin bound to HPA. Insets: Determination of dissociation constant (*K*_*d*_) using one site binding analysis. Linear Van't Hoff plot of azadiradione (C) and gedunin (D) bound to HPA.

Ligand binding studies with fluorescence and CD spectroscopy of azadiradione and gedunin were performed to gain an insight in the mechanism of ligand interaction with the protein. A concentration dependent decrease in fluorescence intensity at 350 nm on binding of azadiradione and gedunin to HPA was observed (Fig 6). *ΔF* represents the decrease in fluorescence intensity relative to the fluorescence intensity of the free enzyme on ligand binding. The unbound HPA (16.6 μg) with activity of 0.25 U ml^-1^exhibited a fluorescence intensity of 193 a.u. at λ_max_ emission of 350 nm. Titration of this enzyme with azadiradione (3.9–118 μM) resulted in quenching of the intrinsic fluorescence to 128 a.u. with maximal quenching of 33% (Fig 7A) and that with gedunin (2.0–51.86 μM) resulted in quenching of the intrinsic fluorescence to 156 a.u. with maximal quenching of 19% (Fig 7B). No emission was noted in the same region for either of the lead inhibitor molecules under any of the abovementioned conditions. Calculation of binding parameters such as the dissociation and association constants (*K*_*d*_ and *K*_*a*_) were performed by linear as well as non-linear regression analysis [40, 41]. Fig 7A and 7B inset shows the non-linear fits for titration at 25°C for azadiradione, gedunin with R^2^ values of 0.95 and 0.975, respectively.

#### Circular Dichroism studies

**Fig 8:**
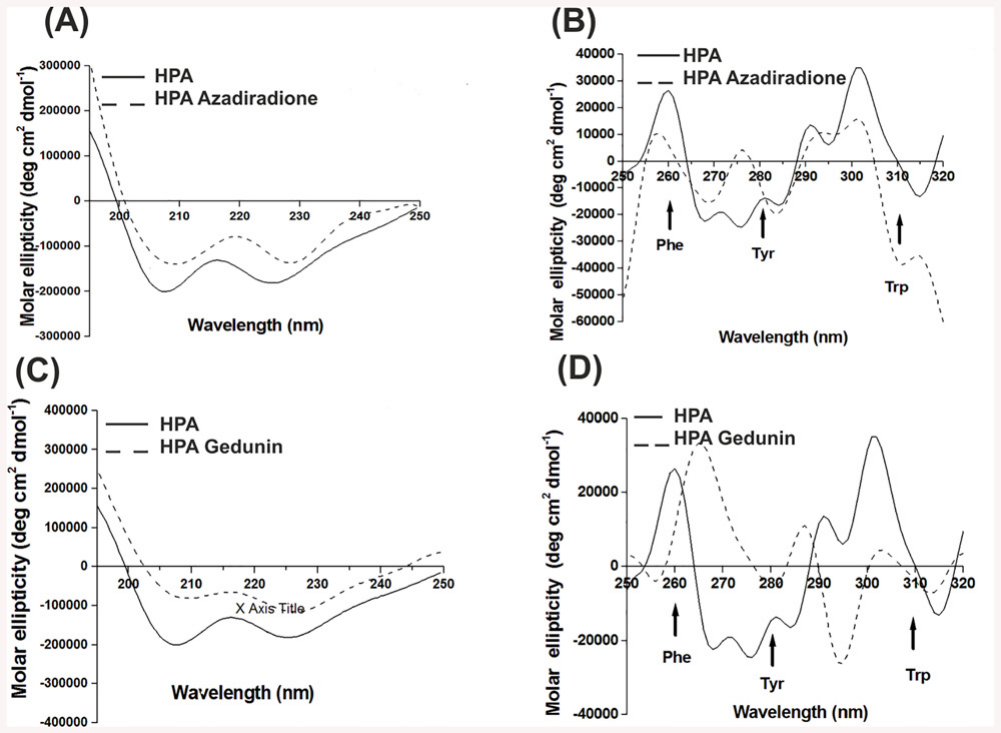
Ligand binding studies by CD spectral analysis. CD spectra of HPA bound with azadiradione (A) Far UV region, (B) Near UV region and gedunin (C) Far UV region, (D) Near UV region.

The ligand binding studies with CD spectroscopy showed the effect of azadiradione and gedunin binding on secondary and tertiary structure of HPA (Fig 8). The far UV-CD spectra (195–250 nm) for HPA in this study were in accordance with the reported spectra for mammalian amylases [42,43].Though a small change in the molar ellipticity was observed in the far UV range, no significant change in the secondary structural elements was observed on binding of either azadiradione or gedunin to HPA (Fig 8A and 8B). The CD spectrum of a protein in the near-UV spectral region (250–350 nm) is sensitive to perturbations of tertiary structure (Fig 8C and 8D). The major changes in spectra of HPA can be attributed to the change in environment of tyrosine at 280, phenylalanine at 260 nm and tryptophan at 290–310 nm. No significant change in the environment of Phe was noted on binding azadiradione to HPA, while a change in the environment of Trp and Tyr was seen. A major shift in the intensity and wavelength occurred on binding of gedunin to HPA with respect to all the aromatic amino acid residues. No significant change in the spectra was noted either in the far-or near-UV region for azadiradione and gedunin alone under the same conditions.

#### Docking of ligands to PPA-oligosaccharide complex

Kinetic studies suggested that azadiradione and gedunin exhibited a mixed mode of inhibition, which led us to dock these ligands on to the enzyme-substrate complex. As mentioned in methods, the PPA-oligosaccharide structural X-ray map obtained from a PPA crystal soaked (and flash-frozen) with a maltopentaose substrate, was taken for docking the limonoids. The PPA-oligosaccharide complex showed an electron density pattern consistent with the binding of maltotriose, the cryotrapped reaction intermediate, from subsites −3 to −1. Another maltotriose, interacting with the K 261 of PPA, protrudes into the solvent indicating the site to be a part of the starch-binding site while two other surface sites were involved in binding of maltose and glucopyranose [39].

**Fig 9:**
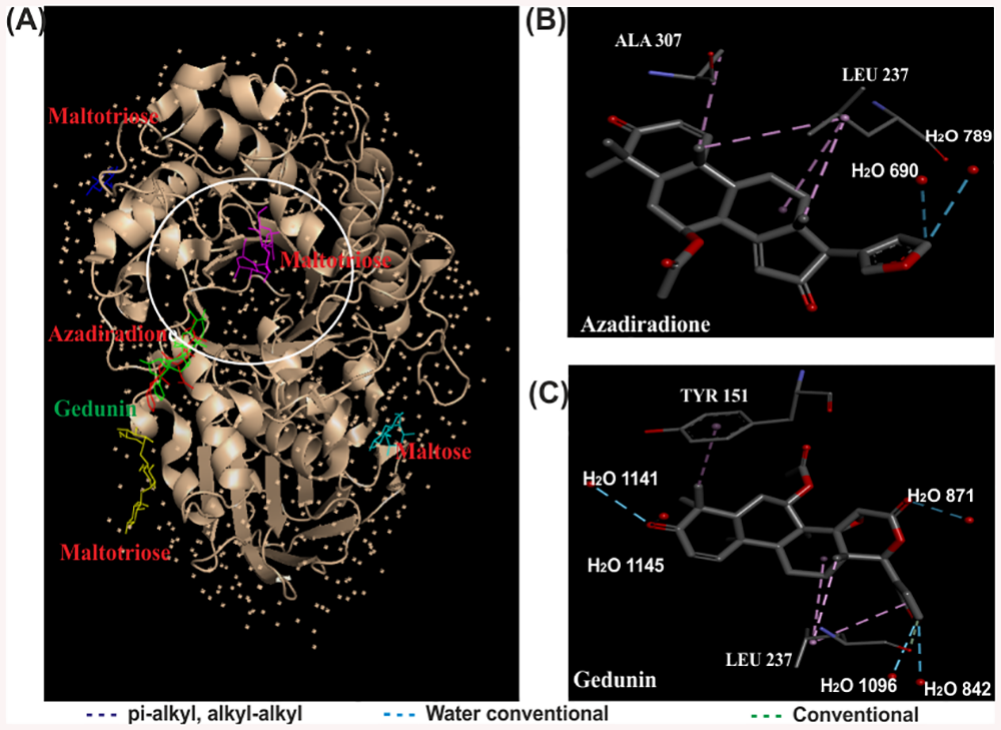
Docking of limonoids to PPA-oligosaccharide complex. Limonoids docked on PPA-oligosaccharide complex (A). Putative mode of interaction between azadiradione, gedunin and with PPA-oligosaccharide complex (B, C).

Blind docking of azadiradione and gedunin to PPA-oligosaccharide complex generated the best pose exhibiting the minimal energy of -24.58 and -25.02 kJ mol^-1^, respectively. The interactions with azadiradione include alkyl-alkyl interaction between Ala 307-C19, Leu 237-C19, Leu 237-C30, Leu 237-aromatic centre of C ring with bond lengths of 4.34, 4.89, 4.41 and 4.76 Å, respectively. Conventional water interactions with a bond length of 3.48 and 3.67 Å was observed between C23 of the furan ring with H2O 690 and H2O 789, respectively. Gedunin forms pi-alkyl interactions between, Tyr 151-C28 of A ring and Leu 237-aromatic ring of furan with a bond length of 4.55 and 4.83 Å, respectively. Alkyl-alkyl interactions are observed between Leu 237-C18 and Leu 237-aromatic centre of C ring with a bond distance of 4.71 and 5.39 Å, respectively. Water conventional interaction was observed H2O 1096-keto oxygen of the furan ring, H2O 842-C23 of furan ring, H2O 1141-C3 keto oxygen of A ring, H2O 1145-C3 keto oxygen of A ring and H2O 871-keto group at C13 position with the bond distances of 3.75, 3.34, 3.24, 3.28 and 3.25 Å, respectively (Fig 9).

### Thermodynamic parameters

The association constants (*K*_*a*_), dissociation constant (*K*_*d*_) and Gibbs free energy (*ΔG°*) of binding were calculated for all six temperatures and provided in (Table 1). The *K*_*a*_ value indicated that the affinity of the ligand for the enzyme decreases on increase in temperature with maximal affinity at 298 K (25°C). The free energy *ΔG°* values calculated from the binding constants are negative, with a maximum *ΔG°* of (-21.25 and -21.16 kJ mol^-1^) for azadiradione and gedunin indicating favorable interactions occurring between HPA and the lead inhibitors. This is in near accordance with the free energy of binding from the docking simulation *i*.*e*. (-25.5, -25.9 kJ mol^-1^) for azadiradione and gedunin respectively. The enthalpy (*ΔH*_*vH*_) and entropy *(-ΔS*) were determined by linear van’t Hoff plot of *K*_*a*_ as a function of temperature with R^2^ of 0.9952 (Fig 7C and 7D). The enthalpy (*ΔHvH*) -41.86 and -139.9 kJ mol^-1^ while the entropy *(-ΔS*) 70.09 and 397.84 J mol^-1^K^-1^were determined for the temperature (298–333 K) for azadiradione and gedunin, respectively suggesting that binding may be both enthalpically and entropically driven.

**Table 1:** Thermodynamic parameters of binding of azadiradione and gedunin to HPA by fluorescence titrations.

	*K*_*a*_(x 10^3^M^-1^)		*K*_*d*_(x 10^-3^M^-1^)		*-ΔG*°(k J mol^-1^)	
Conditions	Azadiradione	Gedunin	Azadiradione	Gedunin	Azadiradione	Gedunin
**298 K**	4.3	5.1	0.2±0.003	0.2±0.009	21.25	21.16
**303 K**	3.9	3.5	0.2±0.001	0.3±0.003	20.88	20.57
**308 K**	2.7	0.6	0.3±0.005	1.6±0.001	20.27	16.4
**313 K**	2.4	0.12	0.4±0.007	8.3±0.05	20.29	12.4
**323K**	1.6	0.08	0.6±0.002	12.2±0.6	19.87	11.8
**333K**	0.81	0.01	1.2±0.03	67.2±0.8	18.54	7.4

## Discussion

Natural products have been known to complement current therapies with lesser side effects to treat DM. Various lead metabolites from hypoglycemic plants such as galegine (*Gallega officinalis*), mycaminose (*Syzium cumini*), kaempferol-3-neohesperidoside (*Bauhinia forficate*), 2’,4’-dihydroxy-4-methoxydihydrochalcone, 4,5-di-O-caffeoylquinic acid (*Artemisia dracunculus L*), quercetin 3-(6-malonylglucoside), rutin, isoquercitrin (*Morus alba*), caffeic acid, p-coumaric acid (*Ocimum sanctum*), curcumin, demethoxycurcumin bisdemethoxycurcumin (*Curcuma longa*), diosgenin from *Dioscorea bulbifera* have been isolated and characterized against various antidiabetic targets [44–47]. Interestingly, all the parts of the evergreen tree *A*. *indica* have been used as traditional medicines in ayurveda and day-to-day household remedies such as anti-inflammatory, hypoglycemic, anti-malarial, anti-ulcer, anti-microbial, anti-carcinogenic, antioxidant and so on. However, very few reports are available on the individual secondary metabolites or their mode of action for hypoglycemic property [48–53].This prompted us to ascertain their efficacy for therapeutic applications as inhibitors of HPA, a potent antidiabetic target.

Careful comparison of α-amylase inhibitory potency of limonoids azadirachtin A, azadirachtin B, salannin, nimbin, azadirone, azadiradione, epoxyazadiradione, gedunin and 17β-hydroxyazadiradione revealed that bioactivity was strictly restricted to the basic limonoid skeleton and no activity was observed with limonoids possessing C-seco type architecture. Variation in activity was observed within the basic limonoid family depending on the presence of skeletal functional groups. These five limonoids contained α, β-unsaturated C3 ketone and 4,4-dimethyl moiety in A-ring as a common feature. They also possess critical structural variations in the D-ring, which may play a pivotal role in α-amylase inhibition. It can be seen that gedunin, showing the highest potency against PPA (IC_50_ 72.2 μM) contains a six-membered lactone D-ring with 14,15β epoxy moiety. When this lactone was converted to a five-membered cyclic ketone with an intact epoxy ring, as observed in epoxyazadiradione, the activity was lowered (IC_50_ 100.2 μM). Further replacement of the epoxy ring with unsaturation as in azadiradione, further reduced the inhibitory potency more extensively (IC_50_ 138.4 μM). In absence of 16-keto and 14,15β epoxy group as in azadirone, no activity was observed. Interestingly, insertion of 17β-hydroxyl group as seen in 17β-hydroxyazadiradione resulted in complete loss of activity vis-à-vis azadiradione, which is structurally similar to 17β-hydroxyazadiradione except for the presence of 17β-hydroxyl group. It has also been reported that the tetranortriterpenoid meliacinolin and azadirachtolide from *A*. *indica* exhibited an IC_50_ of 46.74 μgml^-1^ (91.6 μM) and 55.8 μgml^-1^ (94.09 μM) with PPA [25,26].

The low concentrations of azadiradione and gedunin were found to be non-toxic to AR42J cell line while simultaneously effectively inhibiting the secreted α-amylase activity. The starch load test, mimicking the post prandial conditions in cell lines, are promising as they support the rationale in using limonoids as HPA inhibitors. This data obtained is of relevance as the results obtained from this study can be used to design better water-soluble inhibitors and ascertain their efficacy in animal models.

Looking at the interactions between the limonoids and HPA from the docking simulations, it is clear that the A ring C3 keto oxygen and C29 methyl gains importance due to its interaction with H_2_O 641 and Trp 58 respectively in all the three bioactive limonoids (azadiradione, epoxyazadiradione and gedunin). This suggests that any substitution in the A ring may interfere in the limonoid binding to HPA. Further support for this can be seen with the limonoids azadirachtin A, azadirachtin B, salannin, nimbin,where the A ring has been substituted with bulky functional groups have no bioactivity. Moreover, the limonoids bind near the active pocket without involvement of active site residues in their binding to free HPA, whereas they bind to a site distant from the active site pocket, when docked to PPA-oligosaccharide complex. In mixed inhibition, as has been observed by kinetic and CD studies for HPA by limonoids, the inhibitor binds to a site other than the active site changing the enzyme conformation leading to a reduction in activity (*V*_max_). A reduction in HPA activity in presence of limonoids is observed from our experimental data wherein a decrease in *V*_max_ and an increase *K*_m_ has been shown. Also, CD studies suggests perturbations in the tertiary structure of HPA as seen by changes in environment of Tyr and Trp on binding of limonoids. In a docking study between transchalcone and PPA, Trp 59 and Tyr 62 were reported to be the important aromatic amino acid residues in pi-pi interactions involving the two aromatic rings of ligand and side chain [54]. Similarly, in another study, docking simulations of α-amylase inhibitors from *Protomelas virgatus* methanol extract acrylic acid, 11-octadecenoic acid, 9–12 octadecodienoic acid, 6-oactadecynoic acid, hexadecanoic acid and phthalic acid involve interactions between ligand and Trp 59 / Try 62 suggesting these residues to be important residues in binding of inhibitors to PPA [55]. In the current study, we have observed involvement of Trp 58, Trp 59, Tyr 62 and Tyr 151 in interactions of free HPA and PPA-oligosaccharide with the limonoids, respectively.

Stiochiometry of 1:1 implied binding of one inhibitor molecule to one molecule of HPA. A high R^2^ values for the one-site binding fit obtained in ligand binding studies by fluorescence suggest the one-site binding model is in agreement with the stiochiometry of inhibition. A similar stiochiometry has been observed for inactivation of HPA with bisdemethoxycurcumin [56] and PPA with the anthocyanin, Cy3glc [40]. The amylase inhibitor of black (kidney) beans (*Phaseolus vulgaris*) has also reported to form a 1:1 stoichiometric complex with PPA at pH 5.4 [57–59].

Azadiradione and gedunin exhibited a mixed mode if inhibition as observed by kinetic experiments. The docking with free enzyme results suggests that both inhibitors likely bind near the active pocket. However, the active site residues were not involved in their binding. Moreover docking of azadiradione and gedunin to the PPA-oligosaccharide complex suggests that binding of the ligands occurs at the site distant from the active pocket. Thus mixed inhibition would be of relevance for the regulated activity of HPA in order to slowly release glucose and thereby control PPHG. With the reduction in the limonoids concentration, the HPA activity would be regenerated due to the non-covalent association as observed in docking simulation and the reversible equilibrium with the enzyme. Acarbose has been reported to exhibit noncompetitive mode of HPA inhibition with a *K*_*i*_ value of (22 μM) for 2-chloro-4-nitrophenyl-α-maltotrioside as substrate [60].Interestingly, acetone and aqueous extract of *A*. *indica* leaf have been shown previously to exhibit mixed non competitive mode of inhibition with IC_50_ value of 9.15 mgml^-1^and 5.0 mgml^-1^[61]. α- Amylase inhibitor (α-AI) from *Phaseolus vulgaris* is also reported to exhibit mixed inhibition with maltopentaose as substrate with a relatively high *K*_*i*_ of (5x10^4^ μM) [62].

The quenching of tryptophan fluorescence on titration with the ligand and the change in the CD spectrum of a protein in the near-UV spectral region (250–350 nm) on ligand binding are in accordance with the docking simulation results which shows the involvement of Trp 58, 59 and Tyr 62 in azadiradione binding to HPA and Trp 58 in gedunin binding to HPA. The significant change in the environment of tyrosine and phenylalanine in gedunin to HPA is also observed in the docking simulation, where Phe 256 is at a quenching distance of 9.33 Å. Tyr 62 and Tyr 151 present near the active pocket are quenched as the concentration of ligand increases on titration.

Thus, the ligand binding studies by fluorescence, CD and docking simulation were found to be well in accordance and supportive to each other.

The types of main acting force between pharmaceutical molecule and protein could be judged or assessed according to the relative values change in enthalpy and entropy (*ΔH* and *ΔS)*. *ΔH <*0 and *ΔS <*0, indicates the involvement of hydrogen bonds between the inhibitors and HPA, which is evidenced in the docking simulations too. The interaction between HPA and the limonoids is a spontaneous process with free energy decreasing.

In conclusion, results obtained from this study suggest, azadiradione and gedunin to be lead HPA inhibitory molecules. Thus, the hypoglycemic property exhibited by *A*. *indica* could be justified by HPA inhibition as it could be one of the mechanisms of action. Moreover, this comprehensive study scientifically validates these natural products thereby enabling a better insight with respect to their structure- activity relationship. The study gains importance as these limonoids could be used to design better drug candidates in development of newer inhibitors of HPA for controlling starch digestion in order to reduce post-prandial hyperglycemia.
